# 6-Benzyladenine Treatment Maintains Storage Quality of Chinese Flowering Cabbage by Inhibiting Chlorophyll Degradation and Enhancing Antioxidant Capacity

**DOI:** 10.3390/plants12020334

**Published:** 2023-01-11

**Authors:** Ling Zhang, Xueli Shi, Huaxi Hou, Qinyuan Lin, Shijiang Zhu, Guang Wang

**Affiliations:** Guangdong Provincial Key Laboratory of Postharvest Science of Fruits and Vegetables, Engineering Research Center of Southern Horticultural Products Preservation, Ministry of Education, College of Horticulture, South China Agricultural University, Guangzhou 510642, China

**Keywords:** antioxidant, 6-benzyladenine, Chinese flowering cabbage, chlorophyll, leaf yellowing, postharvest

## Abstract

The cytokinin 6-benzyladenine (6-BA) is widely used to regulate the growth of horticultural crops. However, it is not clear how postharvest treatment with 6-BA at various concentrations affects the quality of Chinese flowering cabbage. In this study, harvested Chinese flowering cabbage was foliar sprayed with 6-BA solution at concentrations of 5, 10, 20, 40, and 80 mg·L^−1^. All 6-BA treatments protected the quality of Chinese flowering cabbage during storage, and the treatment with 20 and 40 mg·L^−1^ 6-BA showed the most obvious effect. Treatment with 6-BA reduced leaf yellowing degree and weight loss rate; maintained high chlorophyll *a* and chlorophyll *b* contents; suppressed the declines in ascorbic acid and soluble protein; enhanced antioxidant capacity; and reduced oxidative damage in cabbage leaves. Furthermore, 6-BA treatment upregulated the expression of antioxidant genes and the activities of SOD, POD, and CAT, while inhibiting the expression of senescence-related gene (*BrSAG12*) and chlorophyll catabolic genes (*BrPAO*, *BrPPH*, *BrSGR1*, *BrNYC1*, *BrRCCR*). These results suggest that postharvest 6-BA treatment enhances antioxidant capacity, delays leaf senescence, and inhibits chlorophyll degradation, thereby maintaining the quality of Chinese flowering cabbage during storage. The findings of this study provide a candidate method for preserving Chinese flowering cabbage after harvest.

## 1. Introduction

The Chinese flowering cabbage (*Brassica campestris* L. ssp. *chinensis* var. *utilis* Tsen et Lee) is one of the most important and widely-consumed vegetables in China [1]. It is popular because of its favorable taste and richness of nutrients, including vitamin C, carotenoids, dietary fiber, minerals, glucosinolates, phenolic acids, and flavonoids [2]. However, the storage and transportation of Chinese flowering cabbage are restricted because of rapid leaf yellowing, wilting, and nutrient loss after harvest [3]. The average postharvest loss of Chinese flowering cabbage is 20–40% in the absence of preservation treatment [4]. Deterioration of vegetable quality after harvest is closely associated with leaf senescence and yellowing. It was reported that the leaf yellowing rate of Chinese flowering cabbage was more than 80% after 4 days of storage at 22 °C [5]. Regulating the plant senescence process is an effective way to reduce the quality deterioration of vegetables during storage [6]. Over the past few years, many studies have focused on technologies for delaying leaf senescence and maintaining vegetable quality after harvest. Our previous study found that preharvest treatment with selenium and SNP can effectively reduce leaf yellowing in Chinese flowering cabbage [2]. Oxidative damage induced by endogenous reactive oxygen species (ROS) was shown to be one of the main triggers of leaf senescence [3]. Over-accumulation of ROS may cause oxidative damage to proteins, DNA, and lipids in plant tissues [7]. Furthermore, oxidative damage can accelerate cell apoptosis and deterioration of cell quality. Modulation of ROS metabolism is a potentially effective solution to protect plant quality and reduce postharvest decay [8].

6-benzyladenine (6-BA), a plant growth regulator, has been widely used in the cultivation of various horticultural crops owing to its high efficiency and low cost [9]. Many studies have shown that 6-BA profoundly inhibits lipid peroxidation and loss of membrane integrity in plant tissues, and it also has a positive effect on delaying plant senescence [10]. 6-BA treatment delays ripening and senescence in mango fruit by regulating ROS production and membrane lipid metabolism [11]. Treatment with 6-BA has also been shown to prolong the storage life of dahlia [12], improve postharvest quality of *A. andreanum* ‘Apalai’ flowers [13], and maintain the postharvest quality of some vegetables, such as Chinese chives [14] and broccoli [15]. However, the effects of 6-BA on vegetables vary substantially among plants. The effects of exogenous plant hormones on plant senescence was considered as either negative or positive [16]. The regulation of 6-BA on leaf senescence and storage quality is closely related to the treatment concentration and methods. We speculated that postharvest application of 6-BA in a certain concentration could delay the senescence and maintain the storage quality of Chinese flowering cabbage. Nevertheless, there have been no studies on the effects of various 6-BA concentrations on Chinese flowering cabbages after harvest. The effects of 6-BA treatment on postharvest quality and related mechanisms need to be comprehensively elucidated.

In this study, we evaluated the effects of different 6-BA concentrations on the postharvest quality of Chinese flowering cabbage. We investigated the effects of 6-BA treatment on the degree of leaf yellowing, weight loss, pigment degradation, nutritional quality, and antioxidant enzyme activity during storage. Moreover, we evaluated the relative expression of chlorophyll metabolic and antioxidant-related genes in postharvest cabbage leaves. The findings of this study explicate the effects of 6-BA treatment on the postharvest quality of Chinese flowering cabbage and provide a candidate method for the preservation of leafy vegetables.

## 2. Results

### 2.1. Postharvest 6-BA Treatment Reduces Weight Loss of Chinese Flowering Cabbage during Storage

As the leaf surface is large, Chinese flowering cabbage is prone to water loss, and weight loss gradually increases with time during the storage period. The results showed that the weight loss rate in the control group increased from 5.81% on day 2 to 6.45% on day 4, and reached 8.75% on day 6 of storage. However, the weight loss rates in the various 6-BA treatment groups were all lower than that of the control at each time point of storage (Table 1). The lowest weight loss rate of postharvest Chinese flowering cabbage was observed in the 40 mg·L^−1^ 6-BA treatment group, which showed weight loss of 2.70%, 3.35%, and 4.42% on days 2, 4, and 6 of storage, respectively. The results suggest that postharvest treatment with 6-BA can effectively improve water retention in Chinese flowering cabbage during storage.

### 2.2. Postharvest 6-BA Trezatment Reduces Deterioration of Appearance Quality of Chinese Flowering Cabbage during Storage

**Figure 1:**
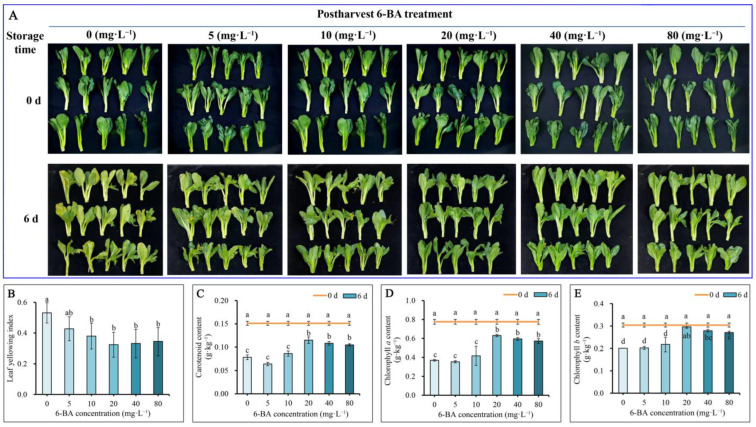
Effects of 6-benzyladenine (6-BA) treatment on appearance quality and changes in pigments of Chinese flowering cabbage during storage. The appearance quality of Chinese flowering cabbage with postharvest treatment of 6-BA at various concentrations (**A**). Leaf yellowing index (**B**), carotenoids (**C**), chlorophyll *a* (**D**), and chlorophyll *b* (**E**) contents of leaf of Chinese flowering cabbage stored at 25 °C for 6 d in the dark. Each bar indicates the mean ± standard deviation of three biological replicates, and different lowercase letters indicate significant differences between Chinese flowering cabbage under different treatments (*p* < 0.05).

Chinese flowering cabbage is prone to wilting and yellowing after harvest. The color of the cabbage leaves changed from dark green to yellow-green during storage (Figure 1A). The leaves in the control group (0 mg·L^−1^ 6-BA) showed obvious wilting, yellowing, and a loose structure. However, the leaf appearance in all 6-BA treatment groups (5, 10, 20, 40, and 80 mg·L^−1^) was greener and fresher than that in the control. Both 20 mg·L^−1^ and 40 mg·L^−1^ 6-BA treatments showed the best appearance quality compared to the control and other treatments. The suppression of leaf wilt and the maintenance of a fresh appearance in 6-BA treatment groups after harvest was consistent with the changes in weight loss (Table 1).

Postharvest leaf yellowing, which becomes severe over time, is one of the most important factors limiting the shelf life of Chinese flowering cabbages. The results showed that the leaf yellowing index values of Chinese flowering cabbage in the 6-BA treatment groups were all significantly lower than that of the control on 6 d of storage (Figure 1B). The carotenoid content (Figure 1C), chlorophyll *a* (Figure 1D), and chlorophyll *b* (Figure 1E) contents in the leaves of Chinese flowering cabbage treated with 6-BA were all higher than those of the control. Moreover, the degradation of leaf pigments in Chinese flowering cabbage during postharvest storage was significantly reduced with increasing 6-BA treatment concentration. The 20 mg·L^−1^ 6-BA treatment showed the best protective effect in maintaining pigment content in cabbage leaves during storage. These results indicate that postharvest treatment with 6-BA maintains the appearance quality of cabbages during storage.

### 2.3. Postharvest 6-BA Treatment Maintains Pigment Content

To further elucidate the profiles of pigment loss, the loss rate of individual pigment content in Chinese flowering cabbage with 6-BA treatments at 6 d relative to 0 d of storage was also studied (Table 2). The results showed that the loss rates of chlorophyll *a*, chlorophyll *b*, and carotenoids in the control group were 52%, 34%, and 48%, respectively. However, the loss rates of these pigments in the 6-BA treatment groups (10 mg·L^−1^, 20 mg·L^−1^, 40 mg·L^−1^, and 80 mg·L^−1^) were lower than those in the control group. In 6-BA treatment groups (20 mg·L^−1^, 40 mg·L^−1^ and 80 mg·L^−1^), the loss rates of chlorophyll *a* and chlorophyll *b* were far lower than those of carotenoids. This finding indicates that 6-BA treatment reduced the degree of yellowing of Chinese flowering cabbage during storage by reducing the degradation of pigments, especially chlorophyll *a* and *b*. The most significant protective effect was observed in the 20 mg·L^−1^ 6-BA treatment group. These results were consistent with the leaf yellowing index and appearance quality results, as described above (Figure 1).

### 2.4. Postharvest 6-BA Treatment Suppressed Senescence Process and Regulated Chlorophyll Metabolism of Chinese Flowering Cabbage

**Figure 2:**
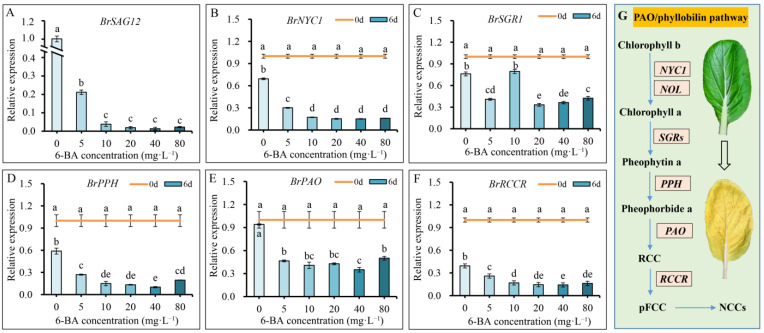
Chlorophyll degradation-related gene expression in Chinese flowering cabbage treated with different 6-BA after harvest. Changes in the transcript levels of *BrSAG12* (**A**), *BrNYC1* (**B**), *BrSGR1* (**C**), *BrPPH* (**D**), *BrPAO* (**E**), and *BrRCCR* (**F**). The PAO/phyllobilin pathway of chlorophyll degradation in plant leaf (**G**). Each bar indicates the mean ± standard deviation of three biological replicates, and different lowercase letters indicate significant differences between Chinese flowering cabbage under different treatments (*p* < 0.05).

Leaves of Chinese flowering cabbage are prone to senescence after harvest, and senescence-associated gene 12 (*SAG12*) is a critical gene promoting senescence progress [3]. The results here showed that *BrSAG12* in the 6-BA treatment group was inhibited compared to that in the control (Figure 2A). The inhibitory effect of 6-BA on the relative expression of *BrSAG12* was enhanced with increasing treatment concentrations, suggesting that the senescence process of cabbages was suppressed by 6-BA treatment. These results were consistent with the appearance quality and degree of leaf yellowing in Chinese flowering cabbage during storage (Figure 1).

Chlorophyll degradation is one of the main metabolic processes that occur during leaf senescence, which is regulated by many chlorophyll catabolic-related genes. The results of this study showed that genes of *BrNYC1*, *BrSGR1*, *BrPPH*, *BrPAO*, and *BrRCCR* were all significantly suppressed by postharvest treatment with various concentrations of 6-BA (Figure 2B–F). These genes have been proved to be critical genes involved in the chlorophyll degradation pathway in plant leaves (Figure 2G) [17]. Treatments of 6-BA at 20 mg·L^−1^ and 40 mg·L^−1^ showed the most significant inhibitory effect on the relative expression of chlorophyll degradation-related genes. These results were highly correlated with the changes in chlorophyll contents, as described above (Figure 1, Table 2). The results suggest that postharvest 6-BA treatment effectively reduced the leaf yellowing by slowing the senescence process and inhibiting chlorophyll degradation in postharvest Chinese flowering cabbage.

### 2.5. Postharvest 6-BA Treatment Maintains Nutrient Value of Chinese Flowering Cabbage during Storage

**Figure 3:**
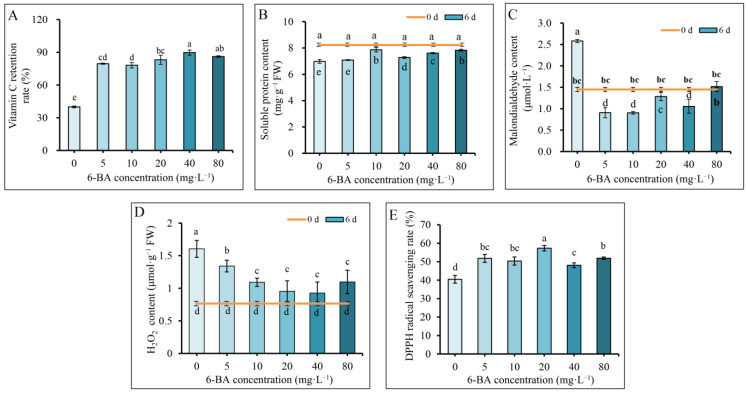
Postharvest 6-BA treatment maintains nutritional quality and antioxidant capacity of Chinese flowering cabbage during storage. Effect of postharvest 6-BA treatments on Vc retention rate (**A**), soluble protein content (**B**), malondialdehyde (MDA) content (**C**), H_2_O_2_ content (**D**), and DPPH radical scavenging rate (**E**) of Chinese flowering cabbage. Each bar indicates the mean ± standard deviation of three biological replicates, and different lowercase letters indicate significant differences between Chinese flowering cabbage under different treatments (*p* < 0.05).

In addition to leaf yellowing, nutritional quality often deteriorates during plant senescence. The results here showed that the Vc retention rate and the soluble protein content at 6 d compared to 0 d of storage varied substantially between the 6-BA treatments and the control group (Figure 3A,B). The Vc retention rates in 6-BA-treated Chinese flowering cabbage were all more than two times higher than that in the control. The best protective effect was found in the 40 mg·L^−1^ 6-BA treatment, which retained 90% of the Vc in the leaf after 6 d of storage (Figure 3A). The soluble protein content in Chinese flowering cabbage leaves was not significantly affected by a low concentration of 6-BA solution (5 mg·L^−1^), but was promoted by the 6-BA treatments at 10 mg·L^−1^, 20 mg·L^−1^, 40 mg·L^−1^, and 80 mg·L^−1^. These results indicate that postharvest 6-BA treatment partly maintained the nutritional quality of Chinese flowering cabbage during storage.

During plant senescence, quality deterioration is usually accompanied by overaccumulation of ROS in tissue, which may in turn cause oxidative damage and accelerate leaf senescence in plants. The malondialdehyde (MDA) level in the leaves can be used as an index to evaluate the degree of oxidative damage in plant [18]. In the present study, the MDA content in the leaves of the control group sharply increased after six days of storage (Figure 3C), which is accompanied by the increase of H_2_O_2_ content (Figure 3D). However, 6-BA treatments suppressed the increase of the H_2_O_2_ content, and the MDA content in the 6-BA treatment groups was significantly lower than that in the control group. Moreover, the antioxidant capacity assay result showed that the DPPH radical scavenging abilities in 6-BA treatments were all stronger than that of the control (Figure 3E). Among these results, the 20 and 40 mg·L^−1^ 6-BA treatments showed better protective effects than the other treatments. These results suggest that postharvest 6-BA treatment effectively reduces oxidative damage and protected storage quality of cabbage during storage.

### 2.6. Postharvest 6-BA Treatment Reduces Oxidative Damage Involved in Regulation of Antioxidant System in Chinese Flowering Cabbage during Storage

**Figure 4:**
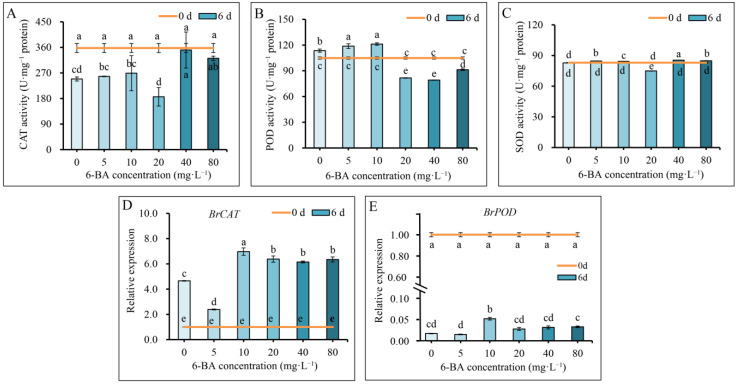
Postharvest 6-BA treatment regulates antioxidant enzyme activities of Chinese flowering cabbage during storage. Effect of postharvest 6-BA treatments on CAT, POD, and SOD activities (**A**–**C**) and the relative expression of *BrCAT* and *BrPOD* genes (**D**,**E**) of Chinese flowering cabbage. Each bar indicates the mean ± standard deviation of three biological replicates, and different lowercase letters indicate significant differences between Chinese flowering cabbage under different treatments (*p* < 0.05).

We investigated the activities of antioxidant enzymes CAT, POD, and SOD during storage. The results showed that CAT activity in the 40 mg·L^−1^ and 80 mg·L^−1^ 6-BA treatment groups was significantly higher than that in the control (Figure 4A). In general, POD activity in Chinese flowering cabbage was enhanced by low concentrations of 6-BA (5 mg·L^−1^ and 10 mg·L^−1^) but suppressed by higher concentrations of 6-BA (20, 40, and 80 mg·L^−1^) (Figure 4B). As for SOD activity, 5 mg·L^−1^, 10 mg·L^−1^, 40 mg·L^−1^, and 80 mg·L^−1^ 6-BA treatments enhanced SOD activity when compared to the control (Figure 4C). These results indicate that 6-BA treatment significantly enhanced the antioxidant capacity by regulating CAT, POD, and SOD enzymes. These results were verified by qPCR analysis, which showed that the relative expression of *BrCAT* genes in the 6-BA treatment groups was markedly higher than that in the control (Figure 4D,E). The relative expression of *BrPOD* was overall suppressed during senescence and it was only significantly up-regulated by 10 mg·L^−1^ 6-BA treatment groups.

## 3. Discussion

The quality of Chinese flowering cabbage deteriorates rapidly after harvesting, which affects its commodity value and shelf life [3]. Green color and water retention of leaves are important commercial qualities of green leafy vegetables [19]. Many studies have attempted to develop techniques to maintain the postharvest quality and extend the shelf life of leafy vegetables. Plant hormones are ideal candidates for delaying leaf senescence [20]. 6-BA, a cytokinin that has various effects on plant growth and senescence, has been widely used in the cultivation of many horticultural crops. The results of this study showed that the postharvest application of 6-BA effectively alleviated leaf yellowing and wilting, and protected the quality of Chinese flowering cabbage during storage. 6-BA enhanced the antioxidant capacity, suppressed the accumulation of ROS and MDA, and suppressed senescence-related genes in leaves. Furthermore, it inhibited chlorophyll catabolism and maintained higher levels of chlorophyll *a* and *b*. We found that 20 and 40 mg·L^−1^ 6-BA treatments showed the best protective effect in maintaining the quality of Chinese flowering cabbage during storage.

The appearance quality of vegetables is one of the most important factors for consumers. Leaf yellowing is one of the most obvious indicators of postharvest quality deterioration of leafy vegetables [21]. The degree of leaf yellowing is used as a quality parameter for vegetables during transportation and marketing [22]. Chlorophyll is the most important pigment in plant leaves owing to its central role in photosynthesis and green color maintenance [23]. The speed of leaf yellowing is mainly determined by the degradation of chlorophyll in the chloroplasts [24]. Chlorophyll degradation is modulated by the “PAO/phyllobilin pathway (Figure 2G),” which is responsible for many catabolic genes including *NYC1*, *SGRs*, *PPH*, *PAO*, *and RCCR* [17,25]. The results showed that the expression levels of chlorophyll catabolic genes, including *BrNYC1*, *BrSGR1*, *BrPPH*, *BrPAO*, and *BrRCCR*, were significantly inhibited in the 6-BA treatment groups compared to those in the control group (Figure 2B–F). These results indicate that 6-BA treatment sustained the chlorophyll content of Chinese flowering cabbage by suppressing the chlorophyll degradation process. This was verified by the higher chlorophyll content in the 6-BA treatment groups compared with that in the control (Figure 1D,E). Moreover, the results showed that the loss rates of chlorophyll *a* and chlorophyll *b* were far lower than those of carotenoids in 6-BA treatment groups (Table 2). Leaf yellowing is a clear indicator of pigment changes, generally due to the exposure of carotenoid color to chlorophyll breakdown. These results well explain the stay-green phenotype and relatively lower leaf yellowing index of 6-BA-treated Chinese flowering cabbage during storage (Figure 1 A,B).

Leaf yellowing also reflects the senescence degree of Chinese flowering cabbage [24]. Chlorophyll degradation in the plant leaves is mediated by many internal and external factors. Changes in plant hormone levels during leaf senescence may play a critical role in chlorophyll degradation. Studies have shown that a decrease in cytokinin concentration occurs in senescent leaves, and the external application of cytokinin can be used to delay leaf senescence and suppress chlorophyll breakdown in plants [26]. In the present study, 6-BA treatment markedly maintained chlorophyll content and suppressed the expression of the senescence-related gene *BrSAG 12* during storage (Figure 2A). This indicated that 6-BA treatment effectively inhibited senescence in leaves, which could explain the decline in leaf yellowing in Chinese flowering cabbage treated with 6-BA after harvest. These findings are consistent with those of many previous studies of other vegetables. For example, treatment with 6-BA effectively inhibited chlorophyll degradation in broccoli florets [15]. Studies have also shown that the effect of 6-BA on delaying leaf senescence is associated with protection of the chloroplast structure and inhibition of chlorophyll degradation [27].

In addition to preserving chlorophyll content, water retention is essential for maintaining the freshness of leaf tissue. Owing to its large surface area, Chinese flowering cabbage quickly loses water after harvest. However, our results showed that postharvest 6-BA treatment effectively enhanced water retention in cabbage leaves. Postharvest treatment with 20 mg·L^−1^ and 40 mg·L^−1^ 6-BA reduced the weight loss rate at 6 d of storage by 42.74% and 49.48%, respectively, compared with the control group (Table 1). Studies have shown that weight loss in postharvest plants is largely due to the acceleration of plant respiration and transpiration during senescence [28]. The enhancement of respiration and transpiration in plants is generally companied by the acceleration of plant senescence [29]. However, the present study showed that postharvest 6-BA treatment markedly suppressed the expression of senescence-related genes *BrSAG 12* in cabbage leaf during storage (Figure 2A). This results well explained the decline in weight loss in Chinese flowering cabbage treated by 6-BA after harvest.

Plant senescence can be induced by internal and external biotic and/or abiotic stressors [30]. Reactive oxygen species (ROS) such as singlet oxygen and superoxide (O_2_.^−^) and hydrogen peroxide (H_2_O_2_) are triggers of senescence in plants [31]. In recent years, the role of ROS in the senescence of postharvest vegetables has received extensive attention [32]. The over-accumulation of ROS in plants during senescence impairs tissues, such as lipids, proteins, and nucleic acids, which increases the MDA content and permeability of the plasma membrane in plants [18]. The quality of vegetables rapidly deteriorates with ROS accumulation in the senescent tissues. The most significant changes in vegetables during storage are a decrease in protein and vitamin C and an increase in MDA in leaves [33]. Soluble protein and vitamin C are important appraisal indices for assessing the nutrition and quality of vegetables [34]. Moreover, soluble proteins are involved in the regulation of various physiological and biochemical metabolic processes in fruits and vegetables, and vitamin C is also an essential antioxidant molecule in plants [35]. Changes in these parameters are often used as indicators to evaluate the quality deterioration and oxidative damage of plant tissues. A previous study showed that ROS and MDA levels in the leaves of Chinese flowering cabbage gradually increase over time during senescence [3]. In this study, 6-BA treatment at 10, 20, 40, and 80 mg·L^−1^ significantly delayed the decline in soluble proteins in the leaves of Chinese flowering cabbage. Cabbage treated with various concentrations of 6-BA (5, 10, 20, 40, and 80 mg·L^−1^) also maintained a higher ascorbic acid content than the control. Moreover, the accumulations of H_2_O_2_ and MDA in the 6-BA treatment groups were significantly lower than that in the control group at 6 d of storage (Figure 3C,D). These results are consistent with previous studies showing that ascorbic acid and soluble protein contents in strawberry and cucumber fruit were elevated by 6-BA treatment. The MDA content in mangoes and pointed gourd (*Trichosanthes dioica* Roxb.) fruits were reduced by 6-BA treatment [11,36]. These results suggest that 6-BA treatment protects the nutritional quality of cabbage against ROS damage during leaf senescence.

**Figure 5:**
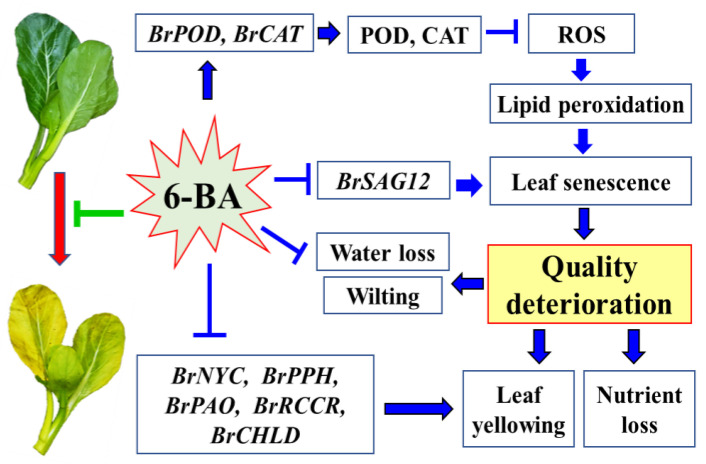
A proposed model of mechanism underlying postharvest 6-BA treatment maintains postharvest Chinese flowering cabbage quality.

In plants, there are corresponding defense systems to reduce the damage caused by ROS, which consist of antioxidant enzymes and non-enzymatic compounds. Antioxidant enzymes, including SOD, POD, and CAT, play an important role in removing ROS from tissues during plant senescence [37]. During the storage period, CAT decreased gradually, while POD and SOD activities in cabbage leaves increased sharply [24]. In this study, the CAT and SOD activities of Chinese flowering cabbage leaves treated with 40 mg·L^−1^ and 80 mg·L^−1^ 6-BA were significantly higher than those of the control leaves. However, the POD activity was increased by the low concentrations of 6-BA treatment (Figure 4A–C). These results were verified by qPCR, which showed that the expression of *BrCAT* genes increased in Chinese flowering cabbage leaves treated with 6-BA at various concentrations (10, 20, 40, and 80 mg·L^−1^), and the relative expression of *BrPOD* was up-regulated by 10 mg·L^−1^ of 6-BA (Figure 4 D,E). The findings here were consistent with the effects of 6-BA on other vegetables and fruits. For example, the activities of SOD, CAT, and APX in 6-BA-treated broccoli were significantly higher, while the MDA content was relatively lower than that of the control [15]. Maintaining cellular redox homeostasis in tissues is beneficial for delaying leaf senescence and alleviating quality deterioration in vegetables [38]. It is reasonable to speculate that 6-BA treatment maintained the balance of ROS metabolism, suppressed lipid peroxidation, slowed down the senescence process, and reduced the quality deterioration of Chinese flowering cabbage during storage (Figure 5).

The quality of harvested leafy vegetable deteriorates rapidly over time. They are prone to leaf yellowing, wilting, and nutrients degradation during storage and transportation [2]. This greatly reduces the nutritional value and commodity value, leading to a huge postharvest loss in leafy vegetable every year [6]. In the present study, we showed that postharvest 6-BA treatment effectively maintains the quality of Chinese flowering cabbage during storage. It is speculated that 6-BA treatment postponed the leaf senescence process, suppressed the chlorophyll degradation, and enhanced the antioxidant capacity during storage. The findings of the present study suggested that 6-BA may have broad application prospects in horticultural crops, especially leafy vegetables and foliage plants. These findings will facilitate the development of new methods to prolong the shelf-life and reduce postharvest loss of horticulture crops.

## 4. Materials and Methods

### 4.1. Materials and Postharvest 6-BA Treatment

Chinese flowering cabbage was purchased from a farmers’ market in Tianhe District, Guangzhou, China. Cabbages with good appearance quality, no yellow leaves, and no mechanical damage were selected for postharvest treatment of 6-BA trials. Thirty plants were randomly selected for each treatment. Different concentrations of 6-BA solution (5, 10, 20, 40, or 80 mg·L^−1^) were sprayed on the leaves of the cabbages. The volume of 6-BA for each treatment group was 50 mL. Control cabbages were sprayed with 50 mL of pure water (0 mg·L^−1^ 6-BA). After air-drying for 2 h, the cabbages were packaged in polyethylene film bags (0.03 mm thickness, with nine holes of 0.5 cm in diameter at each side) and stored in the dark at 25 °C. The day of treatment was considered day 0 of storage, and the cabbages were stored for a further 6 days. The cabbages were weighed at 0, 2, 4, and 6 days of storage, and the cabbages were photographed before and after storage to observe the changes in appearance quality. Changes in nutritional composition and physiological indices were compared between 0 d and 6 d of storage.

### 4.2. Determination of Weight Loss Rate

The fresh weight of Chinese flowering cabbages subjected to the different treatments was measured using a 0.01 g accuracy scale every two days during storage. Weight loss was calculated by subtracting the cabbage fresh weights at 2, 4, and 6 days from those at 0 days of storage, and the weight loss rate was expressed as a percentage of their initial weight at 0 days of storage [2].

### 4.3. Evaluation of Leaf Yellowing Index and Pigments Content

The leaf yellowing index was evaluated using a five-point scale as described in our previous report. The assay of chlorophyll *a*, chlorophyll *b*, and carotenoid contents in cabbage leaves was performed as previously reported [3].

### 4.4. Determination of the Retention Rate of Vitamin C

Vitamin C content (Vc) was measured according to the method described in the previous report [2], with slight modifications. Briefly, cabbage leaves were ground to a powder using liquid nitrogen, and the sample powder (1.0 g) was then extracted with oxalic acid-EDTA solution (3.0 mL). The mixture was centrifuged at 10,000× *g* for 3 min at 4 °C and the supernatant was collected. The reaction was conducted by mixing 1.0 mL of the supernatant, 3.5 mL of oxalic acid-EDTA, 0.5 mL of acetic acid metaphosphate, 1 mL of 5% (*v*/*v*) sulfuric acid, and 2.0 mL of 5% (*w*/*v*) ammonium molybdate. After the reaction, the absorbance of the mixture was measured at 705 nm using a UV-2450 spectrophotometer (Shimadzu, Tokyo, Japan). The vitamin C retention rate after 6 d of storage was calculated as a percentage of the initial content after 0 d of storage.

### 4.5. Measurement of Malondialdehyde (MDA) Content

Leaf samples of Chinese flowering cabbage (1.0 g) were extracted with 10 mL of 5% trichloroacetic acid and centrifuged at 8000× *g* for 10 min at 4 °C. The supernatant was collected and mixed with 1.0 mL of 0.67% (*v*/*v*) thiobarbituric acid, then the mixture was boiled in a water bath for 30 min. After cooling, the absorbance was measured at 532, 600, and 450 nm. The concentration of MDA in the extract was calculated using the following formula: MDA (μmol·L^−1^) = 6.45 × (A532 − A600) − 0.56 × A450 [39].

### 4.6. Determination of the H_2_O_2_ Content and Total Antioxidant Capacity

The H_2_O_2_ content in cabbage leaves was determined by spectrophotometry, and the total antioxidant capacity was evaluated by 2,2-diphenil-1-picrylhydrazyl radical scavenging capacity (DPPH) assay, as described in our previous reports [40].

### 4.7. Measurement the Activities of Antioxidant Enzymes

Powdered Chinese flowering cabbage leaf (1.0 g) was mixed with 5.0 mL of phosphate buffered saline (PBS) extracting buffer containing 2.0 mM β-mercaptoethanol, 1.0 mM EDTA, and 1% (*w*/*v*) polyvinylpyrrolidone (PVP). After centrifugation at 10,000× *g* for 15 min at 4 °C, the supernatant was collected as enzyme extract for evaluating the activities of POD, CAT, and SOD. POD activity was assayed using a reaction system containing 0.1 mL of enzyme extract, 1.0 mL of 50 mmol·L^−1^ pH 7.0 PBS, 1.0 mL of 0.3% H_2_O_2_, and 0.9 mL of 0.2% guaiacol. The absorbance at 470 nm was continuously measured at 1 s intervals for 3 min [41]. CAT activity was evaluated using a reaction system containing 2.9 mL of 0.1% H_2_O_2_ and 0.1 mL of enzyme extract solution [42]. The absorbance at 240 nm was measured at 1 s intervals for 3 min. The change in 0.1 absorbance value per minute per milligram of protein was defined as 1 U. SOD activity was measured according to a previously-described method [41]. The enzyme reaction system contained 1.5 mL PBS (50 mmol·L^−1^ pH 7.8), 0.3 mL methionine (130 mmol·L^−1^), 0.3 mL EDTA-Na_2_ (1 mmol·L^−1^), 0.3 mL riboflavin (0.2 mmol·L^−1^), 0.3 mL nitrogen blue tetrazolium (750 μmol·L^−1^), and 0.3 mL enzyme extract. For the blank, the enzyme extract was replaced with an equivalent amount of water. The absorbance of the mixture was measured at 560 nm after exposure to 4000 lx of light for 8 min. One superoxide disproportionation activity unit (U) was expressed as 50% inhibition of the photoreduction of azotetrazolium blue per minute compared to the blank.

### 4.8. Relative Expression of Genes Assayed by Quantitative Real-Time PCR (qRT-PCR)

Total RNA was extracted from cabbage leaves at the late stage of storage (6 d) using the RNAprep Pure Plant Plus kit (Tiangen, Beijing, China). cDNA was synthesized from RNA using a Hifair III 1st Strand cDNA Synthesis kit and then used as a template for qRT-PCR detection. The relative expression of genes related to the chlorophyll catabolic pathway and antioxidative system was measured using qRT-PCR. The assay was carried out with a Hieff qPCR SYBR Green Master MIX kit (Yi Sheng, Shanghai, China) using a Bio-Rad CFX384 real-time fluorescent quantitative PCR instrument. The qRT-PCR primers used in this study are listed in Table S1. The relative expression levels of the target genes were calculated using the 2^−ΔΔCt^ method with actin as the internal reference [43].

### 4.9. Data Processing and Statistical Analysis

Three replicates were set up for each sample and data were processed using Excel 2010, IBM SPSS Statistics 22, and Origin 2021 for graphing. ANOVA was used to test the significance of differences among treatments.

## 5. Conclusions

In conclusion, our results indicate that postharvest 6-BA treatment at various concentrations can significantly maintain the quality of Chinese flowering cabbage during storage. The treatment with 20 and 40 mg·L^−1^ 6-BA showed the most obvious effect. 6-BA treatment reduces weight loss and leaf yellowing, maintains high chlorophyll *a*, chlorophyll *b*, and carotenoid content in the leaves, and effectively protects the appearance and nutritional quality after harvest. These effects are involved in the modulation of 6-BA in the senescence process and chlorophyll breakdown pathway as well as the regulation of antioxidant capacity. These findings will facilitate the development of new methods to prolong the shelf-life of Chinese flowering cabbage and other leafy vegetables.

## Figures and Tables

**Table 1:** The weight loss rate of Chinese flowering cabbage affected by postharvest treatment of different concentrations of 6-BA.

Storage Time	Weight Loss Rate of Postharvest 6-BA Treatment Groups					
	0 mg·L^−1^	5 mg·L^−1^	10 mg·L^−1^	20 mg·L^−1^	40 mg·L^−1^	80 mg·L^−1^
2 d	5.81 ± 0.09% a	2.85 ± 0.11% c	3.91 ± 0.53% b	3.04 ± 0.19% c	2.70 ± 0.21% c	2.92 ± 0.37% c
4 d	6.45 ± 0.20% a	3.55 ± 0.10% c	4.58 ± 0.55% b	3.82 ± 0.20% c	3.35 ± 0.23% c	3.62 ± 0.39% c
6 d	8.75 ± 0.07% a	4.99 ± 0.34% b	5.71 ± 0.69% b	5.01 ± 0.23% b	4.42 ± 0.20% c	4.76 ± 0.53% bc

The data are expressed as mean ± standard deviation, and different lowercase letters indicate significant differences between different 6-BA treatments at each time point (*p* < 0.05).

**Table 2:** Loss rate of pigment substances at day 6 of storage in Chinese flowering cabbage treated with different concentrations of 6-BA solution.

Pigments	Loss Rate of Pigments in Postharvest 6-BA Treatment Groups					
	0 mg·L^−1^	5 mg·L^−1^	10 mg·L^−1^	20 mg·L^−1^	40 mg·L^−1^	80 mg·L^−1^
Chlorophyll *a*	52 ± 1.06% a	54 ± 1.51% a	46 ± 4.01% b	19 ± 1.61% d	23 ± 1.54% c	26 ± 1.84% c
Chlorophyll *b*	34 ± 1.40% a	34 ± 2.14% a	28 ± 3.31% b	3 ± 0.86% e	8 ± 0.53% d	11 ± 0.55% c
Carotenoids	48 ± 3.39% b	58 ± 2.43% a	43 ± 3.72% b	24 ± 1.18% d	28 ± 2.61% c	31 ± 1.62% c

The data are expressed as mean ± standard deviation, and different lowercase letters indicate significant differences between different 6-BA treatments (*p* < 0.05).

## Data Availability

Not applicable.

## Supplementary Materials

The following supporting information can be downloaded at: https://www.mdpi.com/article/10.3390/plants12020334/s1, Table S1: Primers for Quantitative real-time PCR (qRT-PCR).
