# Supplementary material for: 6-Benzyladenine Treatment Maintains Storage Quality of Chinese Flowering Cabbage by Inhibiting Chlorophyll Degradation and Enhancing Antioxidant Capacity
Source: Plants (Basel). 2023 Jan 11;12(2):334. doi: 10.3390/plants12020334 (PMC9865578; doi:10.3390/plants12020334)
Supplement: Supplementary file 1 [file plants-12-00334-s001.zip › plants-2136440-supplementary.pdf]

Table S1. Primers for Quantitative real-time PCR (qRT-PCR)

Gene name	Forward primer (5′-3′)	Reverse primer (5′-3′)
BrActin1	GGAGCTGAGAGATTCCGTTG	GAACCACCACTGAGGACGAT
BrSAG12	CACTGGCGGCTTAACCACTGAA	GAAGATTGGCTGTATCCTACGGC
BrNYC1	CGTTGAGAGGCTGTCCAGTT	TGAAATCAAGCAGCGGCCTA
BrSGR1	GTCCGCTTTGGGAAGCTACT	ATGAGACCACGGAATCGAGC
BrPPH	TATCTGATGCGCGGGTGGAT	TTCCCGACCAATGCTGGACT
BrPAO	CTTAACCAAGCGCGAGATGC	AGAGCTAAACCGGCCAGAAC
BrRCCR	CTCGACCTCCCTCATCGCAA	CGGACAAAGAGAGACGGCGA
BrCAT	GTTTGATCCTGTCCGGTGCG	CTCACGTTCAGACGGCTTGC
BrPOD	CTTAGTCGCGGCAGGGGAAT	TCGACCTCGCAAACTGAGCA
